# Developmental Block and Programmed Cell Death in *Bos indicus* Embryos: Effects of Protein Supplementation Source and Developmental Kinetics

**DOI:** 10.1371/journal.pone.0119463

**Published:** 2015-03-11

**Authors:** Sheila Merlo Garcia, Luciana Simões Rafagnin Marinho, Paula Alvares Lunardelli, Marcelo Marcondes Seneda, Flávio Vieira Meirelles

**Affiliations:** 1 São Paulo University (USP), Faculdade de Zootecnia e Engenharia de Alimentos, Pirassununga, SP, Brazil; 2 State University of Londrina (UEL), Laboratório de Reprodução Animal, DCV, CCA, Londrina, PR, Brazil; University of Florida, UNITED STATES

## Abstract

The aims of this study were to determine if the protein source of the medium influences zebu embryo development and if developmental kinetics, developmental block and programmed cell death are related. The culture medium was supplemented with either fetal calf serum or bovine serum albumin. The embryos were classified as Fast (n = 1,235) or Slow (n = 485) based on the time required to reach the fourth cell cycle (48 h and 90 h post insemination - hpi -, respectively). The Slow group was further separated into two groups: those presenting exactly 4 cells at 48 hpi (Slow/4 cells) and those that reached the fourth cell cycle at 90 hpi (Slow). Blastocyst quality, DNA fragmentation, mitochondrial membrane potential and signs of apoptosis or necrosis were evaluated. The Slow group had higher incidence of developmental block than the Fast group. The embryos supplemented with fetal calf serum had lower quality. DNA fragmentation and mitochondrial membrane potential were absent in embryos at 48 hpi but present at 90 hpi. Early signs of apoptosis were more frequent in the Slow and Slow/4 cell groups than in the Fast group. We concluded that fetal calf serum reduces blastocyst development and quality, but the mechanism appears to be independent of DNA fragmentation. The apoptotic cells detected at 48 hpi reveal a possible mechanism of programmed cell death activation prior to genome activation. The apoptotic cells observed in the slow-developing embryos suggested a relationship between programmed cell death and embryonic developmental kinetics in zebu in vitro-produced embryos.

## Introduction

In vitro embryo production (IVEP) is used in bovine herds around the world. IVEP was initially used as a final option for donor cows that could not establish pregnancies through other means. However, improvement in this technology has enabled it to become a very practical and competitive reproductive tool, and large-scale IVF programs have been successfully implemented [1,2].

Zebu cattle greatly contribute to large-scale IVEP in both milk and beef herds [1,2]. *Bos indicus* cows usually have four-fold more ovarian antral follicles than *Bos taurus* cows, with averages ranging from 18 to 25 recovered oocytes per OPU session [3]. Furthermore, *Bos indicus* breeds are well adapted to regions with warm weather and high humidity and can maintain productivity under stressful conditions.

Although current IVF results for bovine species are considered satisfactory, approximately 60% of fertilized oocytes do not complete the pre-implantation phase. This period is characterized by the cleavage of a one-cell embryo until just beyond the blastocyst stage and represents an extremely dynamic period of embryogenesis. At this point, the embryo must undergo several cell divisions, epigenetic reprogramming, activation of the embryonic genome, compaction, differentiation into two cell types and development of the blastocoel cavity [4,5].

Among these events, the activation of the embryonic genome is particularly demanding. After activation of the embryonic genome, which primarily occurs at the eight- to 16-cell stage in bovines, the embryo becomes dependent on new transcripts produced by the nucleus to continue development [6]. Embryos that fail to accomplish this task do not survive beyond the eight-cell stage; this phenomenon is known as developmental block. At this stage, a low level of DNA fragmentation [7] and rapid development [8] are indicative of embryonic quality.

The addition of fetal calf serum (FCS) to the culture medium has been studied extensively and appears to affect embryo viability. Despite its undefined and variable composition, FCS is commonly used as a component of culture media for IVEP because it may provide higher rates of blastocysts [9]. However, FCS is known to cause adverse effects such as lipid accumulation [10], changes in mitochondrial structure [11], induction of apoptosis [10] and modifications in gene expression [12]. These findings suggest that FCS may be involved in blastomere apoptosis and fragmentation and, possibly, developmental block.

Embryo fragmentation is a morphological feature that has been recognized as a determinant of viability and appears to be highly related to cellular apoptosis [13]. Increased DNA fragmentation may be related to infertility and implantation failure [14].

In the present study, we investigated whether the ability of *Bos indicus* embryos to overcome developmental block and reach the blastocyst stage is influenced by the protein source in the culture medium (FCS or BSA). In addition, we investigated the association between the speed of development, embryonic block and the activation of programmed cell death (PCD) in the first cell cycles. Our hypotheses were as follows: (1) embryos cultured with FCS have higher rates of developmental block, lower quality and reduced probability of reaching the blastocyst stage; (2) slow-developing embryos have higher rates of developmental block, lower quality and a reduced probability of reaching the blastocyst stage; (3) the developmental block induced by FCS leads to DNA fragmentation of eight-cell embryos; and (4) the speed of embryo cleavage is negatively correlated with developmental block and PCD.

FCS decreased blastocyst rates, embryo quality and the number of cell cycles completed. However, there is no evidence that the effects of FCS on embryo development involve DNA breakage. Fast-developing embryos had higher blastocyst rates, lower rates of developmental block and PCD and presented better quality than slow-developing embryos.

## Material and Methods

All chemicals used in this study were purchased from Sigma-Aldrich (St Louis, MO, USA) unless stated otherwise.

### Study 1: Evaluation of the Potential for Development into Blastocysts

#### Experimental Design

**Fig 1:**
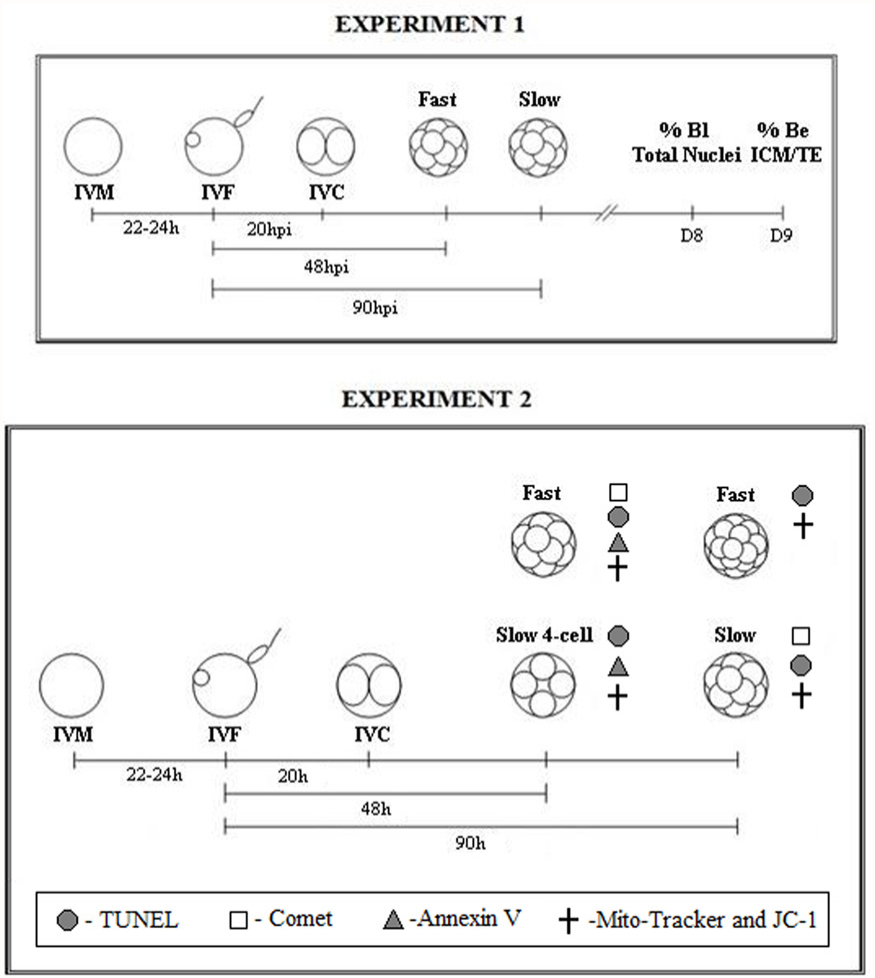
Experimental design. Experiment 1: Oocytes/embryos were subjected to in vitro maturation (IVM), in vitro fertilization (IVF) and in vitro culture (IVC). The embryos were classified according to the time to reach the fourth cell cycle as either Fast (48 h post-insemination—hpi) or Slow (90 hpi). The blastocyst rate (% Bl) and total nuclei count were assessed at eight days post-insemination (D8). To evaluate the hatching rate (% Be) and nuclei count of the inner cell mass (ICM) and trophectoderm (TE) of the hatched embryos were assessed on day 9 (D9). Experiment 2: The Slow group was further separated into two groups: those presenting exactly 4 cells at 48 hpi (Slow/4 cells) and those that reached the fourth cell cycle at 90 hpi (Slow). The cleavage and blastocyst rates, cell number, DNA fragmentation, mitochondrial membrane potential and signs of apoptosis or necrosis were evaluated in the embryos.

The experimental design of Study 1 is illustrated in Fig. 1.

#### Animal Studies

The ovaries used in this study were obtained from a commercial slaughterhouse, from animals that were slaughtered following international guidelines of meat production destined to human consumption. Therefore, ethical approval was not requested.

#### *Ovary Collection and* In Vitro *Maturation (IVM)*

Ovaries from zebu cows were collected at Raja slaughterhouse in Piracicaba, São Paulo, stored in saline solution at 25°C to 30°C and transported to the laboratory. Oocytes with at least three layers of compact cumulus cells and homogeneous cytoplasm were matured in TCM 199 containing Earle’s salts and L-glutamine (Gibco Labs) supplemented with 10% fetal calf serum (FCS; Gibco Life Technologies), sodium pyruvate (22 μg/ml), gentamycin (50 μg/ml), FSH (0.5 μg/ml), LH (0.5 μg/ml) and estradiol (1 μg/ml). IVM was performed in drops of 100 μL covered in mineral oil at 38.5°C and 5% atmospheric CO_2_ for 22 to 24 h.

#### *In Vitro* Fertilization (IVF)

IVF was performed in drops of 100 μL of TALP medium supplemented with 10 μg/mL heparin, 22 μg/ml sodium pyruvate, 50 μg/ml gentamycin, 6 mg/ml fatty acid-free bovine serum albumin (FAF BSA) and PHE (2 μM penicillamine, 1 μM hypotaurine and 0.25 μM epinephrine). Previously tested, frozen-thawed sperm of a Nelore sire kindly offered by CRV Lagoa, Sertãozinho, São Paulo, was centrifuged at 200 X g for 30 min in a 90–45% Percoll gradient. After a visual assessment of motility, the sperm was diluted to a final concentration of 2 x 10^6^ motile sperm/ml. Fertilization was performed in drops in which sperm and oocytes (30 oocytes/drop) were co-cultured for 20 h under the same conditions used for IVM.

#### *In Vitro* Culture (IVC)

After gamete co-incubation, the cumulus cells were removed via successive pipetting. The presumptive zygotes were washed and moved to 100-μL drops (20 embryos per drop) of synthetic oviduct fluid (SOF [15]) supplemented with 10% FCS or 0.8% FAF BSA. In vitro culture was performed in a modular incubator in a low-O_2_ atmosphere (5% CO_2_, 5% O_2_ and 90% N_2_).

At 48 h post-insemination (hpi), the cleavage rate was assessed, and the embryos that were not cleaved were removed. At this time, the culture medium was renewed (feeding). The embryos were separated based on the speed of development: Fast—embryos that reached the fourth cell cycle at 48 hpi and therefore had between five and eight cells; and Slow—embryos that reached the fourth cell cycle at 90 hpi. Embryo development was assessed at days 8 and 9 post-insemination and 383 blastocysts were observed for evaluation of hatching rates. Expanded and hatched blastocysts were used for cell number analysis.

#### Qualitative Embryo Evaluation

For the estimation of cell numbers, 75 expanded D8-blastocysts were fixed in 3.7% paraformaldehyde with 10% Triton X-100 for 1 h and then transferred to a PBS solution supplemented with 0.3% BSA for 1 h. The embryos were stained via immersion in glycerol containing the vital dye Hoechst 33342, and nuclear counting was performed by epifluorescence microscopy.

The hatched D9-embryos (n = 66) were qualitatively analyzed based on the number of cells of the inner cell mass (ICM) and the ratio of ICM and trophectoderm (TE) cells, which was based on differential staining by fluorochrome [16]. The zona pellucida (ZP) was removed from the embryos, and the embryos were washed in TCM-199 Hepes with 10% FCS and in TCM-199 Hepes without FCS. The embryos were then incubated on ice for 10 min in a picric acid solution (10 mM) and polyvinyl pyrrolidone (PVP; 3 mg/ml) in PBS and washed in TCM-199 Hepes. The embryos were subsequently incubated at 39°C for 15 min in an inactivated anti-bovine rabbit serum and diluted 1:10 with TCM-199 medium containing bicarbonate. After washing in TCM-199 medium with 10% FCS, the embryos were incubated at 39°C for 15 min in guinea pig complement diluted 1:10 in TCM-199 Hepes containing 2 μg/ml Hoechst 33342 and 1 μg/ml propidium iodide (PI). Finally, the embryos were washed in PBS containing 0.3% BSA and fixed in blades with glycerol.

The number of live and pyknotic nuclei in the ICM and TE of the embryos was evaluated by epifluorescence microscopy. All nuclei were stained with Hoechst 33342 (vital dye; blue fluorescence). Nuclei with pink fluorescence due to staining with PI (non-vital dye) were considered TE cells and the number of ICM cells was obtained by subtraction.

### Study 2: Evaluation of Developmental block and Apoptosis

#### Experimental Design

To analyze embryonic development block and apoptosis, the embryos were separated based on their speed of development. Those that reached the fourth cell cycle (between 5 and 8 cells) at 48 hpi were classified as Fast; those presenting exactly 4 cells at 48 hpi were classified as Slow/4 cell, and those that reached the fourth cell cycle at 90 hpi were classified as Slow (Fig. 1).

For qualitative analysis, the embryos from the Fast group were evaluated using Comet and TUNEL assays and Annexin V and Mito Tracker/JC-1 staining at 48 hpi. A portion of the Fast group was maintained in culture for up to 90 hpi for evaluation by the TUNEL technique and stained with Mito Tracker and JC-1. The embryos from the Slow/4 cell group were analyzed by TUNEL assay and Annexin V and Mito Tracker/JC-1 staining at 48 hpi. The Slow group was evaluated by the Comet assay at 90 hpi.

#### Comet Assay

Embryos from the Slow (at 90 hpi) and Fast (at 48 hpi) groups were subjected to the Comet assay to detect DNA damage in isolated blastomeres. From the embryos treated with BSA, 58 of the Fast group and 47 of the Slow group were evaluated; from the embryos treated with FCS, 33 of the Fast group and 53 of the Slow group. Embryos were first prepared by removing the ZP with Tyrode’s solution, and the blastomeres were isolated in a solution free of calcium and magnesium. Then, the blastomeres were transferred to a slide with a thin layer of 1% agarose, covered with a low melting point agarose gel [17] and incubated at 50°C for 2 h in lysis solution (10 mM Tris, pH 10, with 2.5 mM NaCl, 100 mM Na2-EDTA, 1% Triton X-100 and 10 μg/ml K proteinase). After 20 min of equilibration in the electrophoresis solution (1 mM Na2-EDTA and 300 mM NaOH), the slides were subjected to electrophoresis for 20 min at 25 V to separate the degraded DNA.

The slides were stained with ethidium bromide, and the DNA damage was assessed by fluorescent microscopy by evaluating the tail length (measured from the cell membrane to the end of the tail) and the proportion of damaged DNA measured using KS 400 software (Carl Zeiss, Inc.).

#### Tunel Assay

In order to evaluate a possible influence of time of culture on DNA fragmentation, the embryos from the Fast and Slow/4 cell groups were analyzed at 48 hpi and at 90 hpi. Of the embryos treated with BSA, 32 from the Fast group and 30 from the Slow group were evaluated; of the embryos treated with FCS, 28 from the Fast group and 18 from the Slow group. The embryos were washed in PBS containing 1 mg/ml PVP and fixed in 3.7% paraformaldehyde for 1 h. The embryos were then permeabilized for 1 h in 0.5% Triton X-100 and 0.1% sodium citrate diluted in PBS and washed in PBS with PVP. The embryos were then incubated in a humidity chamber for 1 h at 37°C in a buffer solution of TDT 10X, CoCl_2_, 2 mM dATP, 0.5 units/μl terminal deoxynucleotidyl transferase enzyme and 0.5 mM Cy3-dUTP and were washed in PBS with PVP.

For DNA visualization by epifluorescence microscopy, slides were prepared by staining with Hoechst 33342 diluted in glycerol (1 μg/ml). The blue fluorescent nuclei (stained using Hoechst 33342) indicated total cell number, and red fluorescent staining (stained using Cy3) indicated cells with fragmented DNA. For each replicate, a few embryos were incubated for 1 h in buffer containing 50 U/ml DNase, thus establishing a positive control group. The results were evaluated based on the number of embryos with more than 50% TUNEL-positive nuclei.

#### Annexin V Staining

The Annexin V (Molecular Probes, Inc.) staining technique enables the differentiation of cell death by apoptosis from cell death by necrosis. In viable cells, phosphatidylserine (PS) is located on the inner surface of the cytoplasmic membrane; in cells in the death process, the PS is displaced from the inner to the outer membrane. Annexin V, which is bound to a fluorescent label, binds to PS in the presence of Ca^+^, enabling its visualization by epifluorescence microscopy. Propidium iodide (PI), a dye that is permeable to damaged membranes and therefore identifies dead cells, assists in distinguishing cells undergoing apoptosis (in which no membrane has been compromised) from necrotic cells. Thus, the results are analyzed as follows: living cells are not stained with PI (neither the cytoplasm nor nucleus); cells undergoing apoptosis are externally stained with Annexin V (blue); and the cytoplasm of necrotic cells is stained with Annexin V, and their nuclei are stained with PI (red). A failure of this method concerns anucleate cells, in which no marking occurs, making it impossible to determine whether they are in the process of apoptosis or necrosis.

The embryos from the Fast and Slow/4 cell groups (both at 48 hpi) were washed in PBS and transferred to the Annexin 1X buffer (200 μL of Annexin 5X buffer in 800 μL of deionized water). Subsequently, they were incubated in a solution of Biotin-X Annexin V (2.5 μL Biotin-X Annexin V in 50 μL 1X Annexin buffer) at 37°C for 45 min. The embryos were then transferred to an Alexa Fluor 350 solution (0.5 μL of 1 mg/ml streptavidin Alexa Fluor 350 in 50 μL of 1X Annexin buffer) for 30 min at 37°C. After this period, the embryos were washed in 1X Annexin buffer and stained with PI (0.2μL of 1 mg/ml PI in 200 μL of Annexin 1X buffer) for 10 min. The embryos were then evaluated by fluorescence microscopy to determine the number of embryos in which more than 50% of cells were stained blue.

#### Mito Tracker Green and JC-1

Changes in membrane potential were qualitatively evaluated using the marker Mitotracker Green as well as JC-1 (Molecular Probes, Inc.) in Fast and Slow/4 cell embryos at 48 and 90 hpi to investigate possible alterations in mitochondrial oxidative phosphorylation. The embryos were incubated with 7.5 μM JC-1 for 40 min and 0.05 μM Mitotracker Green for 30 min and then observed by fluorescence microscopy.

#### Statistical Analysis

For statistical analysis of the cleavage, blastocyst and hatching rates, as well as data obtained using TUNEL and Annexin V techniques, the variable responses were presented as percentage and subjected to a logistic regression test using the Car statistical package of “R” software [18,18]. The average number of cells and differences in the intensity of nuclei damage in the blastomeres (measured using the Comet assay) were presented as mean and standard error and were evaluated by analysis of variance followed by Tukey’s t test (JMP software version 2.0.4; SAS Institute). In Study 1, the number of nuclei at D8 and the hatching rates were conducted separately. In Study 2, the Comet assay, TUNEL, Annexin V and JC-1 were conducted separately. Differences were considered significant when P ≤ 0.05. Changes in the distribution of mitochondria and membrane potential were evaluated qualitatively.

## Results

### Study 1

Embryos supplemented with BSA had higher cleavage (P < 0.01) and blastocyst rates (P < 0.01) than those cultured with FCS (93.5% vs. 84.0% and 31.9/% vs. 26.2%, respectively). Blastocyst rates were higher among Fast embryos than both slow-developing groups in groups cultured with either BSA (54.2% vs. 32.1%; P < 0.01) or FCS (49.4% vs. 31.8%; P < 0.01). There was no significant difference in the blastocyst rates of slow-developing embryos in the groups cultured with different protein sources (32.1% for BSA and 31.8% for FCS; P = 0.910; Table 1).

**Table 1:** Cleavage, blastocyst (8 days post-insemination) and hatching rates (9 days post-insemination) of the Fast and Slow development groups of embryos at the 5–8 cell stage cultured with bovine serum albumin (BSA) or fetal calf serum (FCS) as protein supplements

Protein Source	Oocytes	Cultured	Cleaved		Group	Embryos in the 4^th^ cell cycle		Blastocysts		Hatched blastocysts	
	(n)	(n)	(n)	(%)		(n)	(%)	(n)	(%)	(n)	(%)
**BSA**	1505	1377	1288	93.5^a^	Fast	670	52.0^a^	363	54.2^a^	149/190	78.4^a^
					Slow	240	18.6^b^	77	32.1^b^	23/33	69.7^a^
**FCS**	1448	1364	1146	84.0^b^	Fast	565	49.3^a^	279	49.4^a^	51/131	38.9^b^
					Slow	245	21.4^b^	78	31.8^b^	11/29	37.9^b^

. The blastocyst rates were calculated based on the number of embryos that reached the 4th cell cycle.

a, b—different letters in the same column indicate a significant difference (P ≤ 0.05).

The hatching rate of the BSA group was higher than that of the FCS group for both fast- (78.4% vs. 38.9%; P < 0.01) and slow-developing (69.7% vs. 37.9%; P = 0.01) embryos. However, there were no differences in the hatching rates of Fast and Slow embryos cultured with BSA (P = 0.274) or FCS (P = 0.920).

Of the slow-developing embryos, 68% in both groups (BSA, 32.1% ± 3.0 and FCS, 31.8% ± 3.0) suffered developmental block after reaching the fourth cell cycle. Of the fast-developing embryos, 46% in the BSA group (54.2% ± 1.9) and 51% of those in the FCS group (49.4% ± 2.1) underwent developmental block at this stage.

Approximately 50.7 (1,235/2,434) and 19.9 (485/2,434) % of the cleaved embryos reached the fourth cell cycle at 48 hpi (fast developing group) and 90 hpi (slow-developing group), respectively; the remaining embryos (n = 714) did not reach the fourth cycle prior to 90 hpi.

The blastocysts from the Fast and Slow groups hatched in D9 supplemented with BSA had higher cell numbers than the fast- (181.4 ± 7.1 vs. 126.8 ± 9.0) and slow-developing groups (148.5 ± 12.8 vs. 108.6 ± 9.0) supplemented with FCS (P < 0.01; Table 2).

**Table 2:** Nuclei number of expanded blastocysts 8 days after IVF (Bx—D8) and from hatched blastocysts 9 days after IVF (Be—D9) classified according to the speed of development (Fast or Slow) and sources of protein (BSA or FCS).

ProteinSource	Group	Day 8 Bx nunclei number (mean ± SE)	Day 9 Be nunclei number (mean ± SE)
**BSA**	Fast	134.2 ± 9.1 (24) ^a^	181.4 ± 7.1 (25) ^a^
	Slow	119.4 ± 8.3 (10) ^ab^	148.5 ± 12.8 (14) ^ab^
**FCS**	Fast	124.3 ± 4.5 (30) ^ab^	126.8 ± 9.0 (20) ^b^
	Slow	101.1 ± 7.8 (11) ^b^	108.6 ± 13.8 (7) ^b^

^a, b^—Different letters in the same column indicate significant differences (P ≤ 0.05).

**Fig 2:**
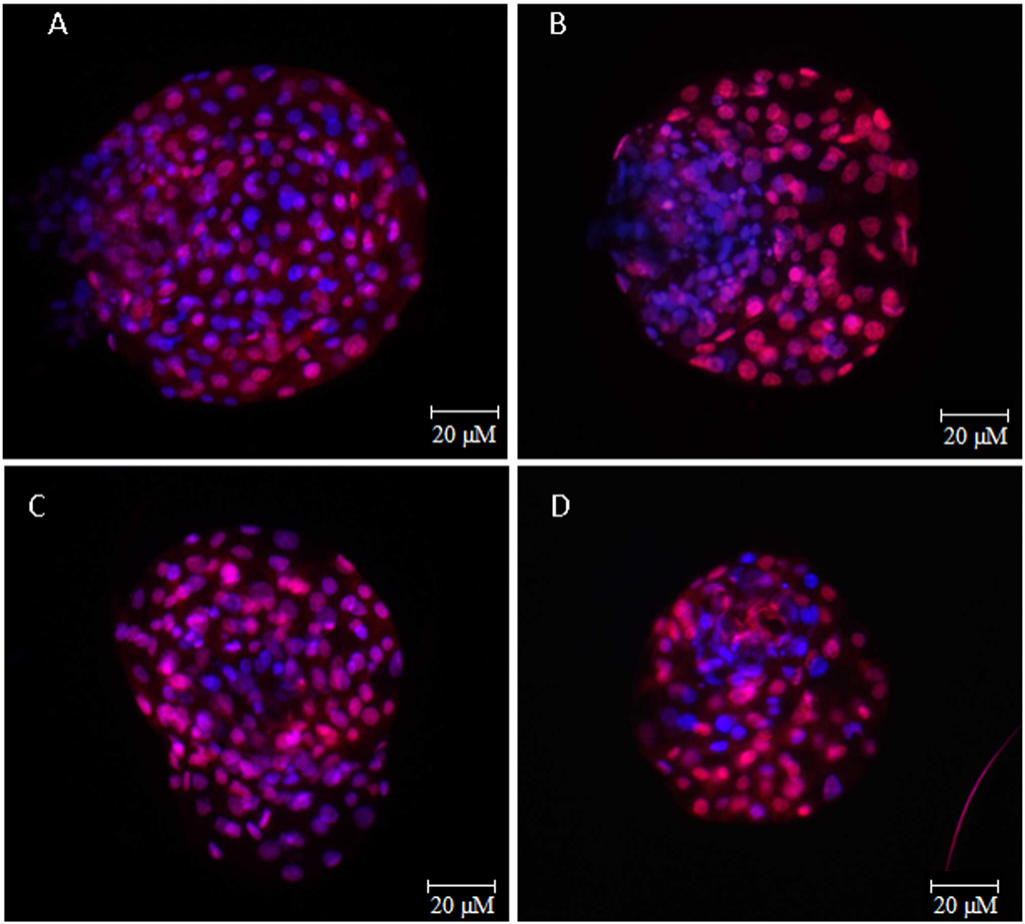
Differential staining by fluorochrome. Epifluorescence photomicrography of the differential staining by fluorochrome of hatched blastocysts cultured with two sources of protein and classified according to the speed of development: A) Fast developing embryo cultured with BSA; B) Slow developing embryo cultured with BSA; C) Fast developing embryo cultured with FCS; and D) Slow developing embryo cultured with FCS.

The embryos supplemented with FCS had fewer cells in the ICM (42.2 ± 3.7) and TE (79.9 ± 5.2) than those supplemented with BSA (58.4 ± 3.4 and 111.2 ± 5.2; P = 0.002 and P < 0.01, respectively; Table 3; Fig. 2). In embryos supplemented with BSA, the Fast group had more cells in the ICM than the Slow group (64.6 ± 4.1 vs. 47.4 ± 4.7; P = 0.012; Table 4). Data regarding nuclei number in the ICM and TE from embryos supplemented with FCS are shown in Table 5. The blastocysts produced with FCS also had more pyknotic nuclei in the ICM than those produced with BSA (5.8 ± 0.8 vs. 3.4 ± 0.4; P = 0.012). However, the ICM/TE ratio remained constant among the treatments (0.56 ± 0.04 and 0.56 ± 0.04 for BSA and FCS, respectively; Table 3).

**Table 3:** Nuclei number in the ICM and TE from hatched blastocysts 9 days after IVF (Be—D9) classified according to the sources of protein (BSA or FCS).

Protein source	N	Nuclei number in the IMC (Mean ± SE)	Pyknotic nuclei number in the ICM (Mean ± SE)	Nuclei number in the TE (Mean ± SE)	Pyknotic nuclei number in the TE (Mean ± SE)	ICM/TE ratio (Mean ± SE)
**BSA**	39	58,4 ± 3,4 ^a^	3,4 ± 0,4 ^a^	111,2 ± 5,2 ^a^	8,0 ± 1,0 ^a^	0,56 ± 0,04 ^a^
**FCS**	27	42,2 ± 3,7 ^b^	5,8 ± 0,8 ^b^	79,9 ± 5,2 ^b^	11,0 ± 1,4 ^a^	0,56 ± 0,05 ^a^

^a, b^—Different letters in the same column indicate significant differences (P ≤ 0.05).

**Table 4:** Nuclei number in the ICM and TE from hatched blastocysts 9 days after IVF (Be—D9), supplemented with BSA, classified according to the speed of development (Fast or Slow).

Speed of development	N	Nuclei number in the IMC (Mean ± SE)	Pyknotic nuclei number in the ICM (Mean ± SE) *	Nuclei number in the TE (Mean ± SE) *	Pyknotic nuclei number in the TE (Mean ± SE) *	ICM/TE ratio (Mean ± SE) *
**Fast**	25	64,6 ± 4,1^a^	3,6 ± 0,5	116,8 ± 4,9	6,9 ± 1,0	0,57 ± 0,03
**Slow**	14	47,4 ±4,7^b^	3,1 ± 0,8	101,8 ± 11,3	9,8 ± 2,3	0,55 ± 0,08

^a,b^—Different letters in the same column indicate significant differences (P ≤ 0.05).

*—There were no significant differences between treatments (P ≤ 0.05).

**Table 5:** Nuclei number in the ICM and TE from hatched blastocysts 9 days after IVF (Be—D9), supplemented with FCS, classified according to the speed of development (Fast or Slow).

Speed of development	N	Nuclei number in the IMC (Mean ± SE) *	Pyknotic nuclei number in the ICM (Mean ± SE) *	Nuclei number in the TE (Mean ± SE) *	Pyknotic nuclei number in the TE (Mean ± SE) *	ICM/TE ratio (Mean ± SE) *
**Fast**	20	45,7 ± 4,5	6,5 ± 1,1	81,1 ± 6,0	11,3 ± 1,7	0,60 ± 0,07
**Slow**	7	32,1 ± 3,7	4,0 ± 0,6	76,4 ± 10,5	10,0 ± 2,1	0,43 ± 0,03

*—There were no significant differences between treatments (P ≤ 0.05).

### Study 2

**Fig 3:**
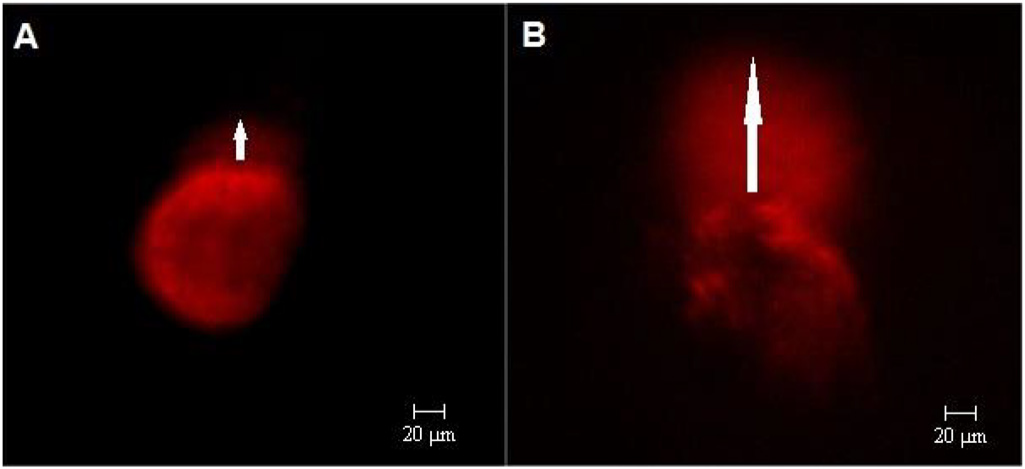
Comet assay. Epifluorescence photomicrography of the results for blastomeres isolated from embryos during the fourth cell cycle, which were classified according to the speed of development as either Fast developing (A) or Slow developing (B). The arrow indicates migrated DNA.

The Comet assay revealed that the Slow group supplemented with either FCS or BSA exhibited significantly higher tail length and degraded DNA density than the Fast group (P < 0.01 in all comparisons; Fig. 3; Table 6).

**Table 6:** Assessment of DNA fragmentation using the Comet assay (tail length and density) and nuclear diameter of the blastomeres of embryos in the fourth cell cycle classified according to the speed of development as Fast (at 48 hpi) or Slow (at 90 hpi) and cultured in media with different sources of protein.

Protein Supplementation	Group	Embryos in the 4^th^ cell cycle (n)	Tail density (%) ± Standard error	Tail length (μm) ± Standard error	Nucleus Diameter (μm) ± Standard error
**BSA**	Fast	58	12.67 ± 1.26 ^b^	27.89 ± 2.55 ^b^	67.65 ± 3.29
	Slow	47	43.38 ± 3.08 ^a^	65.66 ± 5.05 ^a^	63.59 ± 4.09
**FCS**	Fast	33	14.45 ± 2.57 ^b^	23.08 ± 2.72 ^b^	71.83 ± 3.89
	Slow	53	44.72 ± 3.10 ^a^	74.59 ± 5.84 ^a^	70.30 ± 3.31

^a, b^—Different letters in the same column indicate significant differences (P ≤ 0.05).

**Fig 4:**
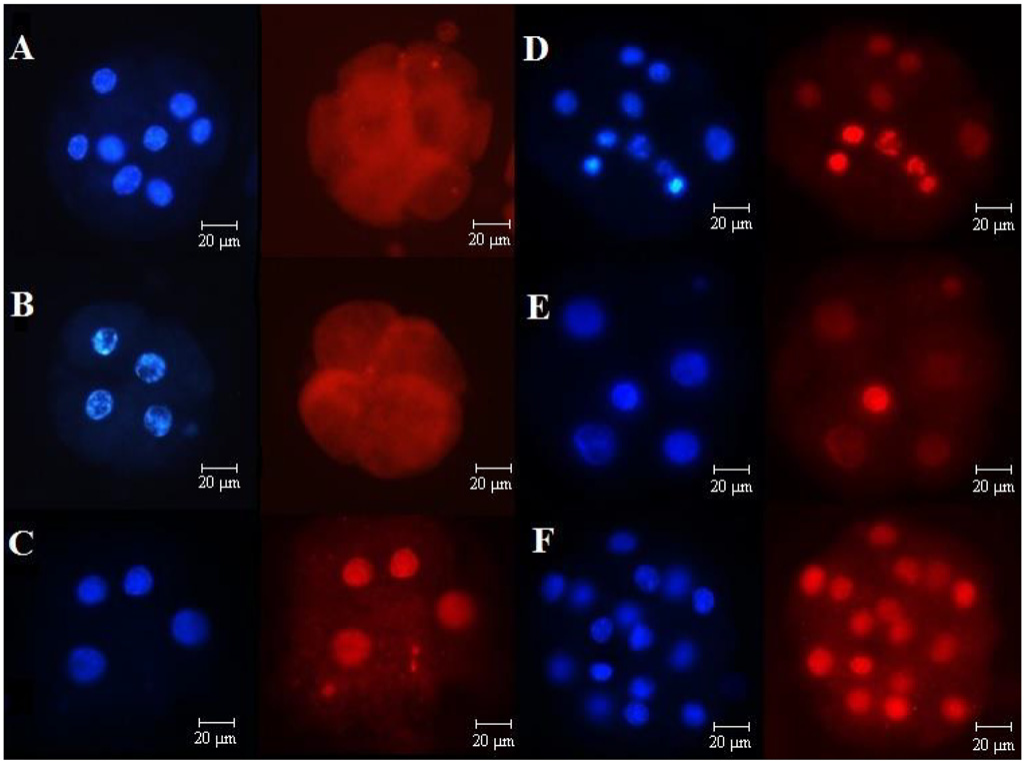
TUNEL assay. Epifluorescence photomicrography of embryos at 48 and 90 hpi classified according to the speed of development as either Fast (reached the fourth cell cycle at 48 hpi) or Slow/4 cells (exactly 4 cells and prior to the fourth cell cycle at 48 hpi). A) Fast embryo at 48 h of culture; B) Slow/4 cell embryo at 48 h of culture; C) embryo at 48 h of culture submitted to DNA fragmentation via exposure to DNase (technique positive control); D) Fast embryo at 90 h of culture; E) Slow/4 cell embryo at 90 h of culture; and F) embryo at 90 h of culture submitted to DNA fragmentation via exposure to DNase. The blue fluorescent nuclei indicate total cell number and red fluorescent staining indicates cells with fragmented DNA.

Fragmented nuclei were not observed by TUNEL assay in any group (Fast and Slow/4 cell supplemented with BSA or FCS) at 48 hpi. However, at 90 hpi, TUNEL-positive nuclei were observed (Fig. 4). In the Fast and Slow/4 cell groups, 25.0% and 41.7% of the embryos, respectively, exhibited greater than 50% TUNEL-positive nuclei (P = 0.068); there were also no significant differences between the embryos supplemented with BSA and those supplemented with FCS (32.3 vs. 32.6%; P = 0.969).

**Fig 5:**
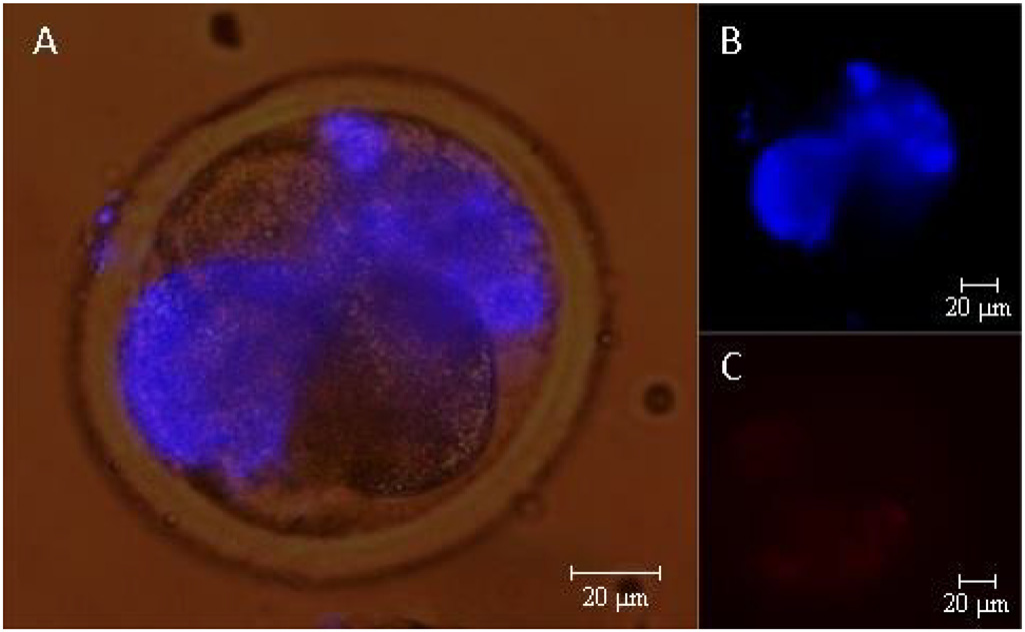
Annexin V staining. Epifluorescence photomicrography of embryos during the fourth cell cycle at 48 hpi: A) embryo with blastomeres undergoing apoptosis; B) blastomeres with blue staining indicating a positive reaction to the Annexin V antibody; and C) blastomeres without membrane permeability to propidium iodide.

The results of Annexin V staining in the Fast and Slow/4 cell groups to assess apoptosis and necrosis are illustrated in Fig. 5. The number of Annexin-positive blastomeres was higher in the Slow/4 cell embryos than the Fast embryos (14.3% vs 1.2%; P < 0.01). There were no differences between the rates of positive cells in the embryos supplemented with BSA (7.9%) and FCS (7.1%). No necrotic nuclei (stained with PI) were observed; however, there were many fragments of anucleated cytoplasm, complicating the results analysis. Among the embryos supplemented with BSA, 15% of the Fast group and 21% of the Slow/4 cell group had at least one anucleated blastomere. Among the embryos supplemented with FCS, 9% and 7% of the Fast and Slow/4 cell groups, respectively, had at least one anucleated blastomere.

**Fig 6:**
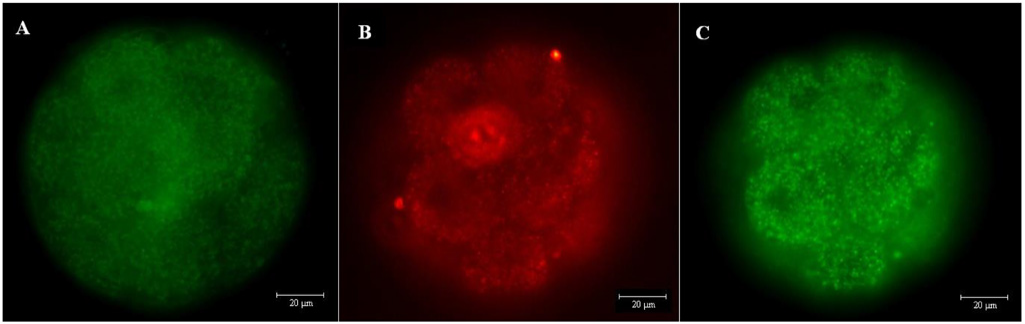
Mitotracker Green and JC-1 staining. Epifluorescence photomicrography of the staining of embryos at 90 hpi: A) blastomeres stained in green = membrane potential detected via staining with Mitotracker Green; B) blastomeres stained in red = membrane potential detected via staining with JC-1; and C) blastomeres stained in green = the absence of membrane potential detected via staining with JC-1.

No membrane potential was detected via JC-1 at 48 hpi regardless of the speed of development. However, the organelles exhibited a low mitochondrial membrane potential, as demonstrated by staining with Mitotracker Green. Both markers indicated the existence of membrane potential at 90 hpi, at which time nuclear fragmentation was also observed (Fig. 6).

## Discussion

In this study, we investigated the influence of the protein source in the culture medium on the ability of bovine embryos to overcome developmental block and reach the blastocyst stage. We also studied the association between embryonic block and the activation of programmed cell death during the first cell cycles. Embryos cultured with FCS had lower blastocyst rates and were of lower quality compared with those produced with BSA. In addition, the speed of development appears to be associated with PCD and developmental block.

The embryos produced with BSA had higher hatching rates than those produced with FCS, indicating that serum influences embryo quality and development capacity. Rooke et al. [20] observed that FCS may alter embryo development in the ovine species; they also reported a biphasic effect in which the inclusion of FCS at the beginning of IVC retarded embryo development. However, the inclusion of serum during the last days of IVC produced a higher blastocyst rate.

Whether supplemented with BSA or FCS, the embryos from the Fast group had higher blastocyst rates than the embryos from the Slow group, which had higher rates of developmental block at the fourth cell cycle. These results are in agreement with previous reports of higher blastocyst rates among fast-developing embryos [19,8]. Slow-cleaving embryos might have a higher incidence of chromosomal abnormalities, altered gene expression and increased DNA double-strand breaks compared to rapidly cleaving embryos [8,7]. In contrast, there are reports of abnormalities in the expression of imprinted genes in fast-developing mice embryos [20]. However, there were no differences in the hatching ability or the ICM/TE ratio among fast- and slow-developing embryos supplemented with either protein source. These results suggest that the developmental blocks at the beginning of embryo development promote selection that results in equivalent potential of the embryos to reach the blastocyst stage in both the fast- and slow-developing groups.

Other studies have also shown that fast-developing embryos present better quality than slow-developing embryos [21,22,23]. It is important to consider that the selection time frame used in the present study was not particularly restrictive. Studies on timing of embryo development performed with time-lapse videosystems provide more detailed information. Somfai et al. [24], using time lapse cinematography, described oocytes showing direct division from one cell to three or four blastomeres, a phenomenon linked to a high frequency of chromosomal abnormalities.

The embryos produced with BSA exhibited increased cell numbers when they developed from expanded to hatched blastocysts. This difference indicates that the embryos from the Fast and Slow groups completed 0.4 and 0.3 cell cycles, respectively, during this period (Table 2). This phenomenon was not observed among embryos supplemented with FCS, which did not exhibit an increase in the number of nuclei from one stage to the next. This lack of increase in the number of nuclei may explain the low hatchability of these embryos, indicating again that FCS leads to lower development capacity and embryonic quality. Another possibility is that embryos supplemented with FCS can accelerate the mechanism of programmed cell death by stimulating factors such as the apoptosis-inducing factor (AIF), which is required for the formation of the blastocoel [25]. Thus, the reduced cell number could be due to the presence of fewer cells at the induction of apoptosis for the formation of the blastocoel. Consequently, the number of remaining cells (that are able to replicate) that are prepared for the opening of the blastocoel cavity would be lower in the embryos treated with FCS. Despite the fact that embryos produced with BSA had higher quality than the embryos produced with serum, it is important to consider that BSA is essentially a protein derived from serum. Therefore, other components of FCS must be harmful for early development of bovine embryos.

The differential staining technique highlights characteristics that are of great importance for embryo survival, such as the ICM/TE ratio and the distribution of morphologically altered nuclei. The embryos supplemented with FCS exhibited lower numbers of total nuclei in the ICM and TE as well as higher number of pyknotic nuclei in the ICM. However, the ICM/TE ratio remained constant among the treatments, indicating that the difference in cell number and embryo quality did not occur due to any effect induced by FCS on differentiation but instead most likely occurred at an earlier stage of development. Alternatively, the total cell number of blastocysts cultured with FCS could be influenced by growth factors. Growth factors can accelerate the mechanism of PCD by stimulating factors such as apoptosis-inducing factor (AIF). Thus, the smaller number of cells reported here could be due to a reduced number of cells available at the induction of apoptosis for the opening of the blastocoel. Consequently, the number of remaining cells (that are able to replicate) would be lower in the embryos that were treated with FCS, which exhibited early induction of cavity formation.

The pyknotic nuclei observed may be related to embryonic cell death; however, this may be a survival and not necessarily a destruction mechanism [26]. The apoptosis of abnormal cells can be a mechanism for the removal of damaged cells and may not be lethal in embryos that also have normal nuclei [27].

Nuclear fragmentation was estimated using the Comet assay, and the Slow group exhibited higher DNA degradation than the Fast group. This DNA damage may reduce the speed of development and increase the developmental block occurring at this phase. The embryos supplemented with BSA or FCS exhibited no difference in the amount of degraded DNA. These data suggest that the effects of FCS are not directly involved in DNA breakage. Serum may cause increased sensitivity to apoptosis induction systems involving cytokines or other constituents, therefore exerting an indirect effect in the blocking of embryo development.

However, the duration of the culture could have exerted an effect on the DNA damage observed in the Comet assay; the embryos of the Fast group reach the fourth cell cycle 48 h after IVF, and the embryos of the Slow group required up to 90 h to reach the same stage. Thus, DNA damage was also analyzed in the Slow/4 cell group using the TUNEL technique before these embryos reached the fourth cell cycle. In this case, the embryos of the Fast and Slow/4 cell groups were not in the same cell cycle and had blastomeres of varying sizes, preventing a comparison of their Comet assays results.

Fragmented nuclei were not observed in any group assessed within 48 hpi; however, a significant increase in the number of TUNEL-positive nuclei was observed at 90 hpi. These data are in accordance with other reports indicating that TUNEL-positive cells are first observed in embryos cultured in vitro between the six- and eight-cell stages [30]. Together, these data indicate that there may be some resistance to nuclear fragmentation during the first three days of culture. Brad et al. [26] reported that the block to apoptosis occurs in two-cell embryos at two points in the apoptotic cascade: at the activation of caspase-9 activity and during caspase-mediated DNA damage.

Annexin V staining was performed to detect early signs of apoptosis (PS exposure) and necrosis (membrane permeability). The exposure of PS observed at 48 hpi may indicate the activation of a cell death mechanism prior to nuclear fragmentation because no DNA fragmentation was detected via the TUNEL assay during the same period. There is limited information in the literature to correlate early embryonic development with PS exposure; however, when combined with other techniques, this could be an interesting tool to understand the mechanism of apoptosis in embryos.

Staining with Mitotracker Green and JC-1 permitted us to assess the changes in membrane potential and also helped us determine whether a change in mitochondrial oxidative phosphorylation of the embryos is associated with DNA fragmentation. The absence of membrane potential at 48 hpi may indicate that low ATP production limits the action of certain enzymes involved in the apoptosis process. After 90 hpi, a mitochondrial membrane potential was verified, which coincided with the appearance of apoptotic nuclei. ATP is necessary for the release of cytochrome C, an enzyme involved in caspase activation that can cause cell death by apoptosis [28]. There is evidence that mitochondria play a central role in regulating programmed cell death and that the release of proapoptotic factors such as cytochrome C and AIF (from the mitochondria) is a primary event in caspase activation [29]. Thus, the absence of membrane potential at 48 hpi could prevent or delay death by apoptosis and could be a mechanism underlying the absence of apoptosis observed via the TUNEL technique in embryos at 48 hpi.

Some of the nuclear fragmentation may have been triggered by oxidative stress caused at the moment of handling the embryos at 48 hpi, when they are subjected to a sudden change in the O_2_ rate. There is evidence that oxidative stress can cause mitochondrial dysfunction [30] and increase DNA damage in embryos [31], thus potentially influencing apoptotic cell death induced by oxidative stress [32].

The specific characteristics of *Bos indicus* breeds must also be considered. In addition to differing numbers of antral follicles, zebu and taurine cows diverge in several aspects, such as estrus manifestation [33], progesterone concentration, follicle size [34] and IGF-I and insulin concentrations [35]. Furthermore, zebu embryos differ from taurine embryos with respect to lipid amount and tolerance to cryopreservation [36]. Therefore, the findings obtained in this study may not extend to *Bos taurus* embryos.

## Conclusions

The results obtained in this study lead us to conclude that, supplementation with 10% FCS during the culture period decreases blastocyst rates and the quality of the embryos produced, thus reducing the number of cell cycles completed as well as hatching ability. Activation of programmed cell death can be detected in early-stage embryos (before the embryos have the ability to perform DNA fragmentation) via Annexin V staining. Lastly, fast-cleaving embryos exhibit higher blastocyst rates, and the speed of development appears to be negatively associated with programmed cell death and the blocking of embryo development.
